# Stem cell applications in regenerative medicine for stress urinary incontinence: A review of effectiveness based on clinical trials

**DOI:** 10.1080/2090598X.2020.1750864

**Published:** 2020-04-17

**Authors:** Bara Barakat, Knut Franke, Samer Schakaki, Sameh Hijazi, Viktoria Hasselhof, Thomas-Alexander Vögeli

**Affiliations:** a Department of Urology and Pediatric Urology, Hospital Viersen, Viersen, Germany; b Department of Urology, Hospital Osnabrück, Osnabruck, Germany; c Department of Urology, Hospital Ibbenbüren, Ibbenbüren, Germany; d St. Elizabeth Boardman Family Medicine, Boardman, OH, USA; e Department of Urology and Pediatric Urology, Universityhospital RWTH Aachen, Aachen, Germany

**Keywords:** Stress urinary incontinence, cell therapy, stem cells

## Abstract

**Objective:**

To evaluate the current state, therapeutic benefit and safety of urethral injection of autologous stem cells for the treatment stress urinary incontinence (SUI).

**Materials and methods:**

A selective database search of PubMed, the Excerpta Medica dataBASE (EMBASE), Cochrane Library and Google Scholar was conducted to validate the effectiveness of stem cell-based therapy. The search included clinical trials published up until 4 January 2020, written in English, and included cohorts of women and men who had received stem cell-based therapy for SUI. The search used the following keywords in various combinations: ‘stem cell therapy’, ‘cell-based therapy for SUI’, ‘regenerative medicine for SUI’, and ‘tissue engineering’. The success rates were assessed according to cough test, urodynamics, pad tests, and International Consultation on Incontinence Questionnaire-Urinary Incontinence. The primary endpoint was continence rate to measure objectively the effect of the treatment.

**Results:**

We identified four clinical trials using local injections of adipose-derived stem cells (ADSCs), 11 trails with muscle-derived stem cells (MDSCs), and two trails with human umbilical cord blood stem cells (HUCBs) and total nucleated cells (TNCs). The median improvement rate of intrinsic sphincter deficiency after ADSCs, MDSCs, TNCs, HUCBs injections were 88%, 77%, 89%, 36% (improvement rate: 1–2 pads) at a mean (range) follow-up of 6 (1–72) months. The cell sources, methods of cell processing, cell number, and implantation techniques differed considerably between studies. Most of the periurethral injections were at the 3, 5, 7, and 9 o’clock positions and for submucosa were at the 4, 6, and 8 o’clock positions. No significant postoperative complications were reported.

**Conclusion:**

Despite many challenges in stem cell-based therapy for treating SUI, it appears to provide, in both male and female patients, acceptable functional results with minimal side-effects and complications. In the future, more clinical trials should be funded in order to optimise stem cell-based therapy and evaluate long-term outcomes.

**Abbreviations:**

ADSC: adipose-derived stem cell; BMSCs: bone marrow-derived mesenchymal stem cell; CLPP: cough leak-point pressure; FPL: functional profile length; HUCB: human umbilical cord blood stem cell; ICIQ-(QOL)(SF)(UI): International Consultation on Incontinence Questionnaire (Quality of life) (-Urinary incontinence Short Form) (-Urinary Incontinence); IIQ-7: Incontinence Impact Questionnaire-short form; I-QOL: Incontinence quality of life questionnaire; ISD: intrinsic urinary sphincter deficiency; MDSC: muscle-derived stem cell; MUCP: maximum urethral closure pressure; NR: not reported; Pdet-max: maximum detrusor pressure; PVR: post-void residual urine volume; Q_max_: maximum urinary flow; QOL: quality of life; RP: radical prostatectomy; TNC: total nucleated cell; (S)UI: (stress) urinary incontinence; UDSCs: urine-derived stem cells; UTUS: upper tract ultrasonography; VLPP: Valsalva leak-point pressure

## Introduction

Urinary incontinence (UI) is a widespread chronic disease and a growing problem, with significant negative impact on the quality of life (QOL) of those affected. It is estimated that >200 million people worldwide are affected by UI [1]. According to recent studies, UI is found in 20–36% of the population aged >40 years [2,3]. Due to the demographic change of an increasingly ageing population, an increase in stress UI (SUI) is to be expected in the future [4]. UI can isolate patients from their professional, sexual, but especially from their social environment. This problem leads to economic and financial burdens, which will increase in the coming years as the age structure of the population changes. The successful treatment of UI requires a pathophysiological understanding of the underlying causes, as well as orienting diagnostics with therapeutic consequences.

SUI can be attributed to different causes, with differences in both sexes. In general, mechanical and functional reasons can be considered as causes of SUI. Important factors are myogenic, neurogenic, connective tissue and hormonal changes. In addition, muscle cell density decreases as a result of physiological apoptosis due to a decrease in the muscle cells of the rhabdosphincter, with a total volume of 88% immediately after birth decreasing to ~34% in the 90th year of life [5]. Female SUI often has a multifactorial cause with functional defects of the urinary bladder sphincter and morphological nerve damage. This is in contrast to the almost exclusively postoperative prostate resection or radical prostatectomy (RP)-related UI seen in men. In recent years, placement of transvaginal tension-free transobturator tape and retropubic tension-free vaginal tape have become well-established treatment options. The mid-urethral sling has the advantage of a shorter duration of intervention time. The rate of any re-operation, including mesh removal, was 5.5% (95% Cl 5.4–5.7%) at 5 years and 6.9% (95% Cl 6.7–7.1%) at 9 years [6]. However, the USA Food and Drug Administration (FDA) has repeatedly issued warnings on the use of alloplastic material in the treatment of female UI due to >1000 reported severe adverse events [7]. Consequently, alternative treatments are being sought and although stem cell-based therapy has had numerous setbacks, it may well be a concept for treating these disorders. In the last decade, the use of the patient’s own adult stem cells for lower urinary tract dysfunction has been shown to be a promising, causal therapeutic approach [8].

## Stem cell therapy

The concept of regenerative medicine is based on the regeneration of the damaged rhabdosphincter, with improvements in the function of the external (striated) muscles and internal (smooth) muscles, as well as the blood circulation of the sphincter [9–12]. Over the last 60 years, significant progress has been made in the field of stem cell and tissue engineering. Stem cells are unique, as they have the potential to differentiate between different cell types and to form a directed population from a single cell.

**Figure 1.**
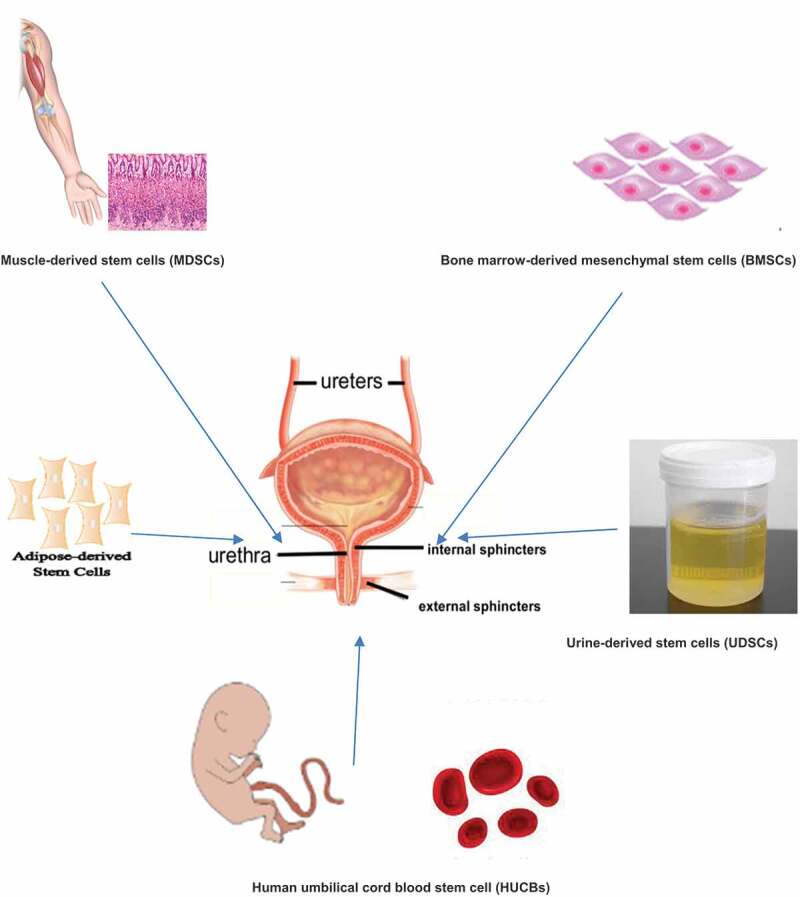
The different sources of stem cell tissues for the treatment of SUI.

Important roles for stem cells include therapeutic, anti-apoptotic, anti-neoplastic and neovascularisation effects [13]. In contrast to embryonic stem cells, adult stem cells have a lower differentiation capacity and a lower proliferation rate [8]. Due to ethical considerations, the blastocyst stage of a human embryo can already be regarded as a human individual, and thus the isolation of embryonic stem cells is prohibited. The extraction and research with adult stem cells is considered ethically harmless. Adult stem cells can be obtained from various sources including haematopoietic [14], epidermal [15], neuronal [16], and mesenchymal [17] stem cells isolated from bone marrow, muscle tissue, adipose tissue [18] (Figure 1), and testicular tissue [19]. Adult stem cells from bone marrow, skeletal muscle, mesenchymal and adipose tissue are ideal candidates for regenerative therapy given the low risk of malignant differentiation, the ability of autologous transfer to eliminate the risk of rejection, and the absence of ethical controversy.

Through the implantation of adult stem cells into the damaged rhabdosphincter, the atrophic, damaged musculature is restored to its correct function through the induction of muscle and nerve regeneration. This is a complex process, which involves the re-modelling of the matrix and the restoration of the cells necessary for proper sphincter-complex functioning and continence. This effect is achieved by prior cell differentiation into striated muscle cells or neurones that can replace the injured structures. The aim of the present review was to summarise the clinical trials, effectiveness and safety of stem cell therapy for the treatment of SUI.

## Patients and methods

### Review criteria

A literature search of PubMed, the National Library of Medicine, the Medical Literature Analysis and Retrieval System Online (MEDLINE), the Excerpta Medica dataBASE (EMBASE), the Cochrane Central Registry of Controlled Trials (CENTRAL), Google Scholar, and ClinicalTrials.gov was undertaken for clinical studies on the treatment of SUI with stem cell therapy. The search included studies published up until 4 January 2020, written in English, and that included cohorts of women and men who had received stem cell-based therapy for UI. The search used the following keywords in various combinations: ‘stem cell therapy’, ‘cell-based therapy for urinary incontinence’, ‘regenerative medicine for urinary incontinence’, and ‘tissue engineering’. In all included studies, the effectiveness of stem cell therapy and continence rate were investigated. The search was limited to clinical studies, fully published articles, systematic reviews, and original papers. Reference lists of articles were also reviewed for relevant articles. The articles were first screened and selected based on their abstracts and then examined in detail. Included articles were selected by consensus of all authors. Two independent researchers reviewed the articles before the final consensus decision was made to include them in the present review.

### Data extraction

The following information was extracted from studies that met the inclusion criteria: name of the first author, year of publication, study design, type of UI, type and number of implanted cells, cell sources, subject data, type of intervention, follow-up data, surgical results, and complications.

### Statistical analysis and data synthesis

The primary outcome of the review was the percentage of continent, partially continent and incontinent patients after surgery. Furthermore, we recorded type and number of implanted cells, cell sources, patient data, type of intervention, follow-up data, outcomes, and complications. Pertaining to UI, outcome measures were summarised based on preoperative UI status, as we considered this the most important factor in postoperative assessment. The treatment outcome was categorised as: ‘healing’, ‘improvement’ or ‘failure’. The ‘objective healing’ was defined as negative cough stress and/or pad testing. ‘Objective improvement’ was defined as the reduction of pads (≥50%). However, if the study did not report the mean and standard deviation (SD), median and sample size were used to estimate the mean and variance. All analyses were performed using the Statistical Analysis Software Comprehensive version 2.0 (Biostat Inc., Englewood, NJ, USA).

## Results of the review

### Study selection, study characteristics and outcomes

**Figure 2.**
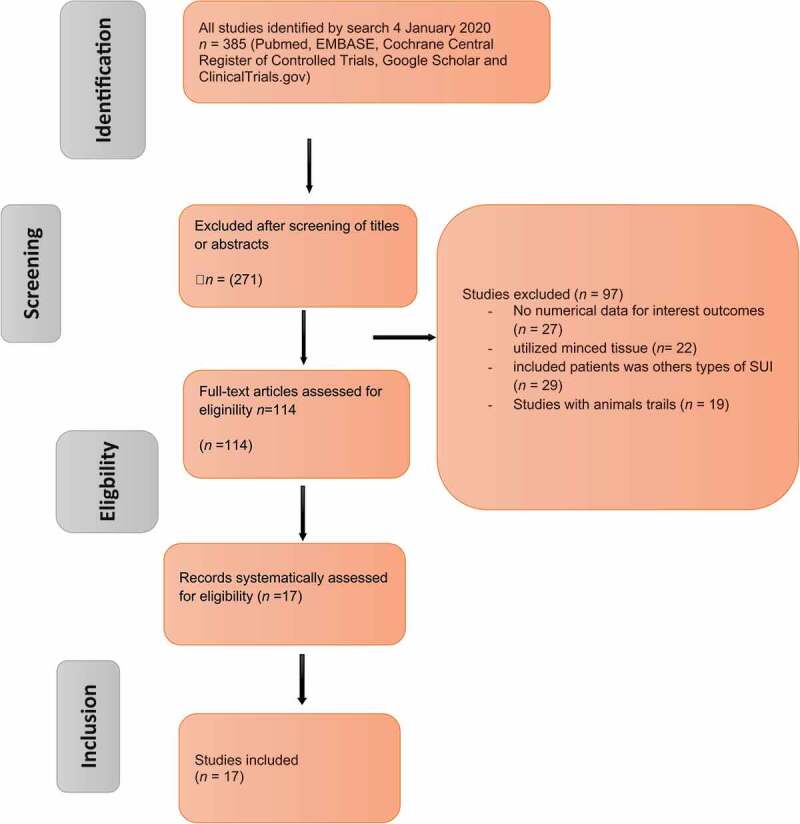
Study selection flow chart, systematic Preferred Reporting Items for Systematic Reviews and Meta-Analyses (PRISMA) search strategy.

The search strategy and the results of the present literature review are shown in Figure 2. After de-duplication, 114 articles were screened for further analysis and 17 studies identified (Tables 1–3 [20,21,22,23,24,25,26,27,28,29,30,31,32,33,34,35,37,32]), which included cohorts of patients undergoing injection of stem cells. In particular, the studies that adhered to the Population, Intervention, Control, Outcome (PICO) quality assessment criteria were included in this analysis. Of the 114 relevant articles, 17 clinical trials (715 patients) met the criteria for inclusion in this analysis (Figure 2, Tables 1–3). The evaluated study designs were prospective analyses of cohorts of patients who underwent injection of stem cells into the urinary sphincter. The number of operating surgeons was not quantified in the included studies. The primary endpoint for the included studies at the last follow-up was continence, categorised as: ‘complete continence’ (no leaking, no pads used), ‘social continence’ (1–2 pads/day), ‘improved UI’ (>50% decrease in number of pads used), or ‘failure’ (<50% improvement, persistent or increased leaking). The secondary endpoint was any complication. The outcomes of included studies were based on urodynamic studies.

**Table 1.** Clinical studies using ADSCs for SUI.

Reference	Patients, *n*	Mean follow-up, months	Continent (no pads) and improvement (1–2 pads), (*n/N*)	Injection site Periurethral injection rhabdosphincter or Submucosa (o’clock positions)	Clinical evaluation	Functional outcomes
Gotoh et al., 2014 [20]	13 Males	1/2, 1, 3, 6, 9, 12, 48	8/11 improvement 3/11 no improvement	5, 7 4, 6, 8	MUCP/FPL/PVR 24-h pad test/ICIQ-QOL/ICIQ-SF	MUCP ↑ (~9.2 cmH_2_O) FPL ↑ (~5.6 mm) PVR ↑ (~4.5 mL)
Choi et al., 2016 [21]	6 Males	3	6/6 improvement	5, 7 4, 6, 8	MUCP/MRI/FPL Pad test/24 h/ICIQ-SF	
Arjmand et al., 2017 [22]	10 Females	1/2, 1,5, 6	10/10 improvement	2, 10	MUCP/FPL/PVR 24-h pad test/ICIQ-QOL/Q_max_	2 weeks (*P* < 0.001) 24 weeks (*P* = 0.018)
Kuismanen et al., 2014 [35]	5 Females	3, 6, 12	3/5 continent 2/5 improvement	3, 9	MUCP/PVR/ 24-h pad test/UDI-6/IIQ-7	NR

FPL: functional profile length; ICIQ-(QOL)(SF)(UI): International Consultation on Incontinence Questionnaire (Quality of life) (Short Form) (Urinary Incontinence); IIQ-7: Incontinence Impact Questionnaire-short form; MUCP: maximum urethral closure pressure; NR: not reported; PVR: post-void residual urine volume; Q_max_: maximum urinary flow; UDI-6: Urogenital Distress Inventory-short form.

**Table 2.** Clinical studies using MDSCs for SUI.

Reference	Patients, *n*	Mean follow-up, months	Continent (no pads), and improvement (1–2 pads), *n/N* (%)	Injection site Periurethral injection rhabdosphincter or Submucosa (o’clock positions)	Clinical evaluation	Functional outcomes
Stangel-Wojcikiewicz et al., 2014 [25]	16 Females	24, 48	8/16 (50) continent 4/16 (25) improvement 4/16 (25)persistent SUI	3, 9, 12	MUCP/CLPP/VLPP	NR
Sharifiaghdas et al., 2016 [26]	10 Females	1, 6, 12, 36	3/10 continent 4/10 Improvement 3/10 persistent SUI	2, 5, 7	MUCP/CLPP/UISS 1-h pad test/IIQ-7 UI score, 24-h voiding diary	MUCP: 15.6 cmH_2_O 12 months: 26.1 cm H_2_O
Peters et al., 2014 [34]	80 Females	1, 3, 6, 12	40/80 (50) Improvement			NR
Cornu et al., 2014 [32]	11 Female same patients as Sebe et al. [32]	72	3/11 (27) improvement	3, 12	24-h pad test	NR
Gräs et al., 2014 [33]	20 Females with complicated SUI 15 Females with uncomplicated SUI		5/20 (25) continent 1/20 (7) improvement 13/20 (63) persistent SUI	3, 9, 12	MUCP/Q_max_/PVR 24-h pad tests/number of leakages in a 3-day diary ICIQ-SF scores	NR
Carr et al., 2013 [23]	38 Female	1, 3, 6, 12, 18	29/38 (76) improvement	External urethral sphincter	24-h pad test/IIQ-7 1-h pad test, IIQ-7/UDI-6	NR
Blaganje et al., 2012 [37]	38 Females	1, 1/2	5/38 (13) continent 29/38 (76) improvement 3/38 (7) persistent SUI	Two levels	Stress test/I-QOL/VAS	Improvement VAS (= 5)/I-QOL (Stress test)
Sebe et al., 2011 [32]	12 Females	1, 3, 6. 12	3/12 continent 7/12 improvement 2/12 worsening	3,9	Q_max_/PVR/24-h pad test	NR
Mitterberger et al., 2007 [24]	123 Females	12	94/119 (79) continent 16/119 (13) improvement 9/119 (8) slight improvement	Mid-urethra	MUCP/VLPP/MUCP MBC/Q_max_/Pdet-max/MRI/24-h pad test/UI score/QOL score	NR
Gerullis et al., 2012 [27]	222 Males	6	26/222 (11) continent 94/222 (42) improvement 102/222 (45) persistent SUI	Around urethra	NR	VLPP/MUCP/MBC/Q_max_/Pdet-max/MRU/24-h voiding diary/pad test: (~5)/QOL (~50) score.
Mitterberger et al., 2008 [28]	63 Males	12	41/63 (65) continent 17/63 (26) improvement 5/63 (8) persistent SUI	Around urethra Around submucosa	NR	VLPP ↑ (~22 cmH_2_O) MUCP ↑ (~17 cmH_2_O) MBC ↑ (~26 mL) Q_max_ ↑ (~2 mL/s) Pdet-max ↑(~8 cmH_2_O) MRU ↑ (~37.5 mL)

CLPP: cough leak-point pressure; FPL: functional profile length; ICIQ-(QOL)(SF)(UI): International Consultation on Incontinence Questionnaire (Quality of life) (Short Form) (Urinary Incontinence); IIQ-7: Incontinence Impact Questionnaire-short form; I-QOL: Incontinence quality of life questionnaire; MBC: maximum bladder capacity; MRU: Maximum residual urine; MUCP: maximum urethral closure pressure; NR: not reported; PVR: post-void residual urine volume; Q_max_: maximum urinary flow; SUI: stress urinary incontinence; UDI-6: Urogenital Distress Inventory-short form; UISS: Urinary inventory stress test; VAS: visual analogue scale; VLPP: Valsalva leak-point pressure.

**Table 3.** Clinical studies using other types of stem cells, HUCBs and TNCs.

Reference	Patients, *n* Stem cell type	Mean follow-up, months	Continent (no pads), and improvement (1–2 pads), *n/N* (%)	Injection site Periurethral injection rhabdosphincter or submucosa (o’clock positions)	Clinical evaluation	Functional outcomes
Lee et al., 2010 [30]	39 Females only 36 completed follow-up Heterologous HUCBs	1, 3,12	13/36 (36) continent 13/36 (36) improvement 10/36 (27) persistent SUI	4,8	MUCP	MUCP/PVR 1-h pad test/cough test/ICIQ-UI/ICIQ-QOL
Shirvan et al., 2013 [31]	9 Females TNCs/platelets	1, 3, 6	8/9 improvement 1/9 persistent SUI	1.5, 3, 4.5, 6, 7.5, 9, 12	MUCP ↑ (~25 cmH_2_O)	↑ MUCP (from <30 cmH_2_O)

ICIQ-(QOL)(UI): International Consultation on Incontinence Questionnaire (Quality of life) (Urinary Incontinence); MUCP: maximum urethral closure pressure; PVR: post-void residual urine volume.

Derived stem cells (MDSCs). Bone marrow-derived mesenchymal stem cells (BMSCs). Urine-derived stem cells (UDSCs). Human umbilical cord blood stem cell (HUCBs)

### Excluded studies

Initially, our search yielded 385 publications. After removal of the duplicates, 271 articles remained and were screened using their title and abstract; leaving 114 articles selected for full-text review. Another 29 studies were excluded from the review because an endpoint (SUI) was not defined and mentioned in the context (*n* = 29) or they utilised minced tissue (*n* = 22). While, 27 studies were excluded because the results were not quantitative outcomes. Another 19 studies were excluded due to being trails in animals. The flow of studies through the selection process is presented in Figure 2.

## Adipose-derived stem cells (ADSCs) implantation

ADSCs are one of the most commonly used stem cell types in autoplastic transplantations. Using ADSCs is advantageous due to the easy recovery of adipose tissue. This has led to large quantities being recovered safely with minimal morbidity [38]. Several studies have shown that ADSCs are multi-potent cells and can differentiate into adipogenic, chondrogenic, myogenic and osteogenic cells [38,39]. All this makes adipose tissue an attractive source for stem cell therapy. Adipose tissue has a high content of ADSCs, with ~15 million ADSCs/g of adipose tissue. In addition, ADSCs have the ability to proliferate rapidly, even at low serum levels. Rodriguez et al. [40] showed that ADSCs can be obtained from processed lipoaspirates and can differentiate into functional urothelial and smooth muscle cells, which can be contracted with cholinergic stimulation. Adipose tissue can therefore be an important cell source for the treatment of injured tissues in which smooth muscle plays an important role. ADSCs therapy is not only used in female UI, but also plays an important role in the treatment and restoration of male UI. Gotoh et al [20], Yamamoto et al [41] and Choi et al. [21] provide evidence that periurethral injection of autologous ADSCs is a safe and technically feasible treatment for men with moderate SUI after RP and holmium laser enucleation of the prostate. In total, we found five clinical trials (40 patients) using adult ADSCs (three in men and two in women). The largest patient scope was the Gotoh et al. [20] study with 13 male patients, showing that leakage volume (in frequency and amount of UI) decreased by 59.8% (from 260.7 to 152.7 g) and improved QOL. They showed that the mean maximum urethral closure pressure (MUCP) increased by 9 cmH_2_O from baseline, the functional profile length (FPL) increased by 6 mm, and the post-void residual urine volume (PVR) decreased after treatment from 52 to 4.5 mL. In addition, they reported an increase in urethral length of 2.2 mm on MRI after injection (Table 1). Choi et al. [21] used ADSCs to treat persistent UI after RP in six men. They showed a decrease in the amount of UI and a significant increase in MUCP by 19.5 cmH_2_O from baseline. The Arjmand et al. [22] study of 10 female patients showed significant short-term improvement in UI after ADSCs injection [2 weeks after injection (*P* < 0.001) and after 24 weeks (*P* = 0.018)]. The interpretation and direct comparison of studies was difficult due to variability in surgical techniques and the number of injected cells. The median postoperative improvement rate was 88% after ADSCs injection (improvement rate: 1–2 pads).

It is worth noting that the overall data situation is not sufficient for assessing the long-term safety of the results. The published studies have small populations and short-term results. Further scientific studies with a longer follow-up period and more patients are required. However, the available results indicate a regenerative potential of ADSCs for the treatment of SUI.

## Muscle-derived stem cells (MDSCs) implantation

Despite technical difficulties in the past, MDSC-based therapy has proven to be a promising option for patients with UI in recent years, with the cultivation of these cells and the selection of stem cells [42]. As such, MDSC-based therapy is an important and easily accessible source of adult stem cells for the treatment of UI. The procurement of muscle tissue is associated with minimal morbidity by the easy sampling of muscle biopsy under local anaesthesia [43]. However, the isolated autologous MDSCs must first be expanded *in vitro* and cultured for a short time before injection into the urethral sphincter [42,43,44]. Deasy et al. [45] and Wu et al. [46] have shown that MDSCs have great potential for tissue regeneration through their proliferation and self-renewal. MDSC-injection therapy offers the advantage that the cells injected into the rhabdosphincter form so-called myotubes and myofibres, which are not only regarded as blocking agents, but also are physiologically capable of improving the function of the sphincters [47]. In all the clinical studies published on UI, autologous MDSCs were injected directly into the rhabdosphincter either transurethrally or periurethrally.

Overall, we identified 11 clinical trials (637 patients) using MDSCs; nine included only female patients (*n* = 352) and two studies included male patients (*n* = 285) (Table 2). The first clinical study of MDSCs (42 women and 21 men) with autologous myoblasts and fibroblasts was conducted by Strasser et al. [48] between 2002 and 2004. The fibroblasts were injected into the submucosa of the urethra, whereas the myoblasts were implanted in the rhabdosphincter. However, it must be mentioned that this clinical study of the Austrian research group was later withdrawn due to scientific irregularities, violations of ethical guidelines, and protocol irregularities. The Austrian research group reported an improvement in QOL and contractility of the urethral sphincter, with a success rate of >90% for women and >50% for men.

The subsequent study by Carr et al. [49] in 2008 was a follow-up at 1 year in eight female patients. Each patient received MDSC-based therapy under local anaesthesia. Five of the eight women showed a realistic improvement in symptoms and one achieved complete continence. The same research group published another study in 2013 with 38 female patients who had been treated with a low MDSCs dose (1, 2, 4, 8 or 16 × 10^6^) and a high MDSCs dose (32, 64 or 128 × 10^6^) [23]. The autologous MDSCs were injected ultrasonically into the sphincter muscle. A total of 32 patients (20 with a low-cell dose and 12 with a high-cell dose) had a significant reduction in the number of wet pads (pad test) at the 3-month follow-up. It should be mentioned that the results in the high-dose group were better than those in the patients treated with a low dose (88.9% vs 61.5%, 77.8% vs 53.3%). In a similar population, the Mitterberger et al. [24] study of 123 female patients, with an average follow-up of 62.8 months, provided evidence of a significant improvement in SUI after injections of myoblasts and fibroblasts in the middle of the urethra. At the 1-year follow-up after implantation of the cells, 94 women (79%) were continent and 16 women (13%) had a significant improvement.

A clinical study by Stangel-Wojcikiewicz et al. [25] in 2014 included 16 female patients and showed an improvement in UI in four patients (25%), at a follow-up of 12 months based on clinical parameters (Gaudenz questionnaire) and urodynamic parameters [cough leak detection pressure (CLPP); Valsalva leak-point pressure (VLPP)]. Eight of the 16 patients (50%) were continent after 8 months (Table 2). In 2016, Sharifiaghdas et al. [26] published their prospective, small cohort study of 10 female patients receiving autologous MDSC therapy for severe SUI. At a follow-up of 36 months, three patients showed full continence based on the cough stress test, 1-h pad test and UI impact questionnaire. Four had significant improvement in UI, and three patients did not respond to the treatment.

In one of the largest clinical trials done, Gerullis et al. [27] obtained four muscles biopsies in 222 patients; 192 with UI following RP, nine after TURP, and 21 after radical cystoprostatectomy with neobladder. The autologous MDSCs were expanded in vitro after successful cultivation and directly injected into the sphincter under endoscopic view in a cell dose of 5.2 × 10^6^. At a follow-up of 6–12 months, only 26 patients (12%) were continent, 94 patients (42%) had improved, and 102 patients (46%) reported persistent UI.

Mitterberger et al. [28] and his team used a mixture of autologous myoblasts and fibroblasts from muscle biopsies in patients with UI after RP. The fibroblasts were suspended in small amounts of collagen as carrier material and injected into the urethral submucosa, while the myoblasts were implanted into the rhabdosphincter. The injection was ultrasonically controlled with a specially developed injection device and injected into the rhabdosphincter (15–18 doses, 50–100 μL/dose) at the 5 and 7 o’clock positions in two different stages. Then, 25–30 doses of fibroblasts/collagen were injected circumferentially into the submucosa. At the 1-year follow-up, 41 men (65%) were completely continent, while 17 (27%) had a significant improvement (Table 2).

The interpretation and direct comparison between clinical trails with MDSCs was difficult due to variability in surgical techniques and the number of injected cells. The median postoperative improvement rate was 77% after MDSCs injection (improvement rate: 1–2 pads).

The studies published using MDSC-based therapy show potential for improving sphincter function by re-modelling damaged sphincter muscle and bulking the urethra with new tissue [29,50]. However, MDSC-injection therapy does have certain disadvantages including migration and absorption of cells, as is the case with other commercial injectable agents. It will require the development of new strategies, such as tissue engineering, to improve the retention and survival of transplanted stem cells.

## Other types of stem cells: bone marrow-derived mesenchymal stem cell (BMSCs), urine-derived stem cells (UDSCs), human umbilical cord blood stem cell (HUCBs), and total nucleated cells (TNCs)

Bone marrow contains heterogeneous cell populations, including erythrocytes, macrophages, endothelial cells, fibroblasts, adipocytes, and haematopoietic/mesenchymal stem cells [51]. BMSCs develop in the bone marrow and can renew themselves and differentiate into different cell types. The first BMSCs were described as osteogenic progenitor cells in 1966. Later, the BMSCs were used in tissue engineering. The procurement of BMSCs can be achieved with sufficient density for stem cell-based therapy. Disadvantageous for the use of BMSCs in clinical use is the painful bone aspiration under local anaesthesia, thus requiring general anaesthesia for the procedure. The isolated BMSCs must also be expanded *in vitro* and cultivated for a short time prior to the final injection. To our knowledge, no BMSCs have been used in clinical urology to date. Some animal model studies have shown that the injected BMSCs were able to restore damaged urethral sphincter tissue and significantly improve UI [52]. However, the results of other animal studies were very contradictory to these findings.

UDSCs can easily be recovered from human urine and expanded in vitro. The advantage of using UDSCs as a cell source for treatment lies in the easy removal without invasive measures. This represents a considerable financial advantage compared to extraction of other cell types by means of biopsy. To the best of our knowledge, few experimental studies using UDSCs for the treatment of UI currently exist. Zhang et al. [53] collected 55 urine samples from 15 healthy individuals and eight patients with VUR. The UDSCs were then successfully isolated and expanded. The authors showed that the individual clones of UDSCs have the ability to differentiate into cell lines that express urothelium, smooth muscle, endothelial and interstitial cell markers [50,53].

Human umbilical cord blood stem cells (HUCBs) can be extracted from human umbilical cord blood. HUCBs are not considered embryonic cells, which is a great advantage compared to other cell sources. Stem cells from elderly people have a low differentiation potential compared to stem cells from young people [54]. Another benefit of using stem cells from umbilical cord blood is the ease of harvesting it without invasive measures. The Lee et al. [30] clinical study is the only one that used HUCBs in 39 women with UI after failure of conservative and surgical therapy. The umbilical cord vein was punctured after normal delivery and human umbilical cord blood was then collected in an aseptic collection bag containing 23 mL citrate-phosphate-dextrose-adenine (CPDA-1) and stored at – 70°C.

Lee et al. [30] injected HUCBs under local anaesthesia into the submucosal area of the proximal urethra at the 4 and 8 o’clock positions (430 ± 190 × 10^6^ cells/2 mL). At the 12-month follow-up, 13 patients (36%) were completely satisfied and 13 patients (36%) had significant improvement of their UI (Table 3). However, 10 patients (27%) in this study remained incontinent. Urodynamic examination at 3 months in 10 of the patients showed that the MUCP almost doubled after cell injection.

Multipotent endothelial cells (EPCs) can be taken from peripheral blood to produce autologous TNCs and used for the treatment of UI. The role of growth factors and the effects of platelet-rich plasma have been investigated with in vitro and in vivo studies. This effect has been associated with several growth factors produced by platelets (platelet-derived growth factor, transforming growth factor β1, fibroblast growth factors, vascular endothelial growth factors, etc.) [54]. We found a single clinical pilot study using platelet-mixed TNCs for the treatment of severe female UI [31]. In that study, 120 mL of peripheral blood was drawn for the separation of platelets and nucleated cells. After centrifugation, the total volume of the platelets and TNCs was equal. A mixture of 10 mL was then injected into the periurethral tissue. Eight of nine patients had a clinically significant improvement according to the International Consultation on Incontinence Questionnaire-Urinary Incontinence (ICIQ-UI) and ICIQ-QOL at the 3- and 6-month follow-ups (Table 3). One patient had an improvement in UI, but was not cured.

## Challenges with stem cells in the treatment of SUI

Although the clinical studies on stem cell therapy for the treatment of SUI show encouraging results with great potential, these short-term results must be interpreted with caution. The results of various clinical trials are controversial. One challenge is the lifespan of stem cells. The reabsorption of autologous fat is rapid. Cell membrane damage is caused by suction and only 10–30% of the adipose cells can be detected 6 months after application. Myeloblasts, theoretically, have the potential to develop functional muscular structures. However, at present it can only be speculated whether the myeloblasts can actually form new fibres or contribute to the contractility of an atrophic sphincter. It is also unclear how the necessary nerve supply of the ‘neosphincter cells’ takes place. The very high success rates in most published clinical studies with very small numbers of patients require critical consideration. After an initial 90%, only 70% success rates are published with filling materials after appropriate selection. Although the filling volume is only ~3–4 mL of substance, an additional bulking effect must be assumed, and thus as persistent obstruction effect could result. The lifespan of implanted stem cells is relatively short. In addition, cell death due to ischaemia, inflammation, or apoptosis may occur within the first week [55]. The size and function of the biotechnological muscle decreases with increasing age [56]. This certainly plays a further role in the lifespan of the stem cells. Furthermore, one of the major challenges for stem cell treatment is not only the selection of stem cells with great differentiation potential, but also optimisation of the technical processes. Some studies were even withdrawn after publication due to ethical and regulatory concerns [32,48].

As shown in Table 2, most clinical studies have been conducted with autologous MDSCs. Treatment with autologous MDSCs requires an optimal lengthy isolation/cultivation work process prior to injection. In all published UI clinical studies, autologous MDSCs were injected either transurethrally or periurethrally directly into the rhabdosphincter, but the number of transplanted cells varied greatly. The different cell doses used are due to the lack of a clear understanding of stem cell-based therapy. However, it is indisputable that the concept of regenerative medicine leads to regeneration of the damaged rhabdosphincter with improvement in function of the external (striated muscle) and internal (smooth muscle) sphincters, as well as the blood circulation of the urethral sphincter.

Both differentiated cell and mesenchymal stem cell therapies have become attractive instruments to improve biocompatibility, tissue integration and minimise adverse inflammatory responses. However, the available studies are very heterogeneous, making it difficult to make comparisons between cell types or cell coating processes. In addition, only a few studies have been conducted in clinically relevant animal models, leading to contradictory results. Finally, a comprehensive understanding of the biological mechanisms of MDSCs associated with the foreign body reaction is required.

## Conclusion

Despite many challenges stem cell-based therapy for treating SUI appears to provide, in both male and female patients, acceptable functional results with minimal side-effects and complications.

In summary, future studies should focus on the following points:

(1) The standardised selection of stem cells.

(2) Compliance with regulatory guidelines for clinical trials.

(3) Establishment of a long-term, clinically relevant animal model with long-term outcomes.

(4) A feasibility study in a multicentre clinical trial with more patients and longer follow-ups.
